# 985. Pooled RNA Metagenomics to Enable Scalable, Unbiased Pathogen Detection and Surveillance

**DOI:** 10.1093/ofid/ofad500.040

**Published:** 2023-11-27

**Authors:** Katelyn S Messer, Gordon Adams, Jillian S Paull, Jonathan Livny, Jacob Lemieux, Daniel J Park, Pardis C Sabeti, Katherine J Siddle

**Affiliations:** Broad Institute of Harvard and MIT, Boston, MA; Massachusetts General Hospital, Boston, Massachusetts; Harvard University, Brighton, Massachusetts; Broad Institute, Cambridge, Massachusetts; Massachusetts General Hospital, Boston, Massachusetts; Broad Institute, Cambridge, Massachusetts; Harvard TH Chan School of Public Health, Cambridge, MA; Brown University, Providence, Rhode Island

## Abstract

**Background:**

As disease patterns shift with changing demographics and climate, developing comprehensive and scalable tools for tracking and identifying emerging pathogens is an increasingly critical component in mounting a robust public health response. Metagenomic next-generation sequencing (mNGS) provides unbiased detection of etiological agents from diverse types of clinical specimen and has great potential to aid in the prediction and prevention of future outbreaks. However, available protocols are unsuitable for widespread and routine use, due to high cost and extended hands-on time required for sample processing. Thus there is a need for faster, cheaper, and more automatable sequencing methods that retain the accuracy and breadth of current protocols.

**Methods:**

Here we develop Pooled RNA Metagenomics; a cost-effective and highly-multiplexed method that accelerates the processing of RNA samples by barcoding individual samples early in the pipeline allowing for parallel preparation. We benchmark this method by comparing the output from clinical samples to that from other established mNGS methods. Additionally, the protocol is designed to preserve the RNA sense information in the resulting sequencing data, and we incorporate unique molecular identifiers upstream of amplification; two features that enhance the utility and flexibility of the method for downstream applications.

Pooled RNA Metagenomics Overview

**Figure:**
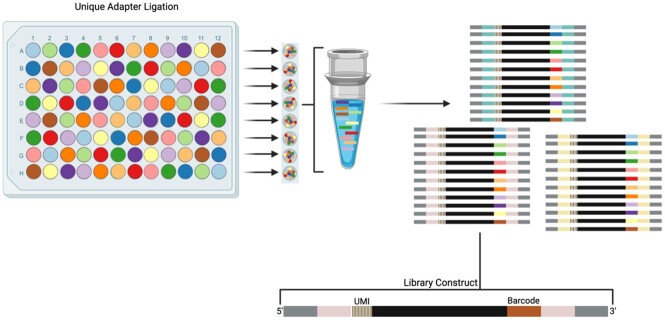

Schematic overview of the pooled mNGS workflow: RNA samples are ligated to uniquely barcoded adapters before being pooled together for cDNA synthesis with UMI incorporation and subsequent library construction PCR.

**Results:**

We found that early pooling of barcoded RNA samples can reduce reagent costs by up to 80% and processing time by up to 60%. We demonstrate that pooling up to 32 samples in a single well results in similar recovery of complete viral genomes to those produced by other mNGS methods where each RNA is processed separately.

**Conclusion:**

Pooled RNA Metagenomics provides an innovative, cost-effective, scalable, and pathogen-agnostic approach to outbreak monitoring and prevention. The high throughput enabled by this method can be augmented by incorporating automation, which in tandem increases speed and scale. The protocol is also amenable to miniaturization, which can further reduce costs and is of particular interest for low input clinical specimens. In summary, Pooled RNA Metagenomics dramatically lowers the barrier to high throughput mNGS, making it a viable option for enhanced pathogen surveillance.

**Disclosures:**

**Pardis C. Sabeti, MD, PhD**, Danaher Corp: Board Member|Danaher Corp: Stocks/Bonds|Delve Bio: Advisor/Consultant|Delve Bio: Stocks/Bonds|NextGen Jane: Advisor/Consultant|Sherlock Biosciences, Inc: Advisor/Consultant|Sherlock Biosciences, Inc: Stocks/Bonds

